# The YfkO Nitroreductase from Bacillus Licheniformis on Gold-Coated Superparamagnetic Nanoparticles: Towards a Novel Directed Enzyme Prodrug Therapy Approach

**DOI:** 10.3390/pharmaceutics13040517

**Published:** 2021-04-09

**Authors:** Patrick Ball, Robert Hobbs, Simon Anderson, Emma Thompson, Vanessa Gwenin, Christopher Von Ruhland, Christopher Gwenin

**Affiliations:** 1 School of Natural Sciences, Bangor University, Bangor, Gwynedd, Wales LL57 2UW, UK; patrick@stonejunction.co.uk (P.B.); r.j.hobbs@swansea.ac.uk (R.H.); simon.a93@hotmail.co.uk (S.A.); thompson.emmaclaire@gmail.com (E.T.); vanessa.gwenin@gmail.com (V.G.); 2 College of Biomedical and Life Sciences, Cardiff University, Cardiff CF14 4XN, UK; VonRuhlandCJ@cardiff.ac.uk; 3 Department of Chemistry, Xi’an Jiaotong-Liverpool University, Suzhou 215123, China

**Keywords:** cancer, nitroreductase, prodrug, CB1954, DEPT

## Abstract

The bacterial nitroreductase NfnB has been the focus of a great deal of research for its use in directed enzyme prodrug therapy in combination with the nitroreductase prodrug CB1954 with this combination of enzyme and prodrug even entering clinical trials. Despite some promising results, there are major limitations to this research, such as the fact that the lowest reported Km for this enzyme far exceeds the maximum dosage of CB1954. Due to these limitations, new enzymes are now being investigated for their potential use in directed enzyme prodrug therapy. One such enzyme that has proved promising is the YfkO nitroreductase from Bacillus Licheniformis. Upon investigation, the YfkO nitroreductase was shown to have a much lower Km (below the maximum dosage) than that of NfnB as well as the fact that when reacting with the prodrug it produces a much more favourable ratio of enzymatic products than NfnB, forming more of the desired 4-hydroxylamine derivative of CB1954.

## 1. Introduction

Cancer chemotherapy is an area of research that is of the utmost importance with cancer being responsible for over 30% of global deaths each year [1,2]. To increase the tumour selectivity of cancer chemotherapy treatments and combat the problem of systematic toxicity, alternative treatment methods being explored involve the use of prodrugs [3,4,5,6,7]. One chemotherapy strategy that is currently being investigated involving the use of prodrugs is directed enzyme prodrug therapy (DEPT), this strategy involves delivering prodrug-activating enzymes directly to a tumour site before administering a prodrug, thereby activating the prodrug to its more pharmaceutically active products at the cancer site [6]. A variety of delivery vectors have been explored for potential DEPT application, including antibodies (ADEPT) [8,9], viruses (VDEPT) [10] and genes (GDEPT) [11,12,13,14]. We are investigating a novel form of DEPT that utilizes gold coated magnetic nanoparticles (AuMNP) as the delivery vector for genetically modified prodrug activating enzymes called magnetic nanoparticle directed enzyme prodrug therapy (MNDEPT) [15,16]. 

**Figure 1:**
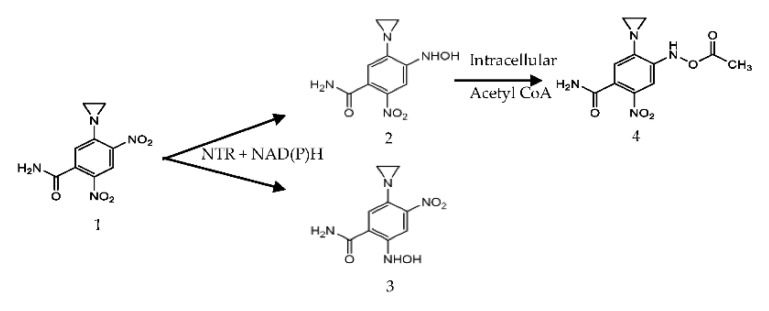
The reduction of CB1954 (**1**) into its products: 5-(aziridin-1-yl)-4-(hydroxyamino)-2-nitrobenzamide (2) 5-(aziridin-1-yl)-2-(hydroxyamino)-4-nitrobenzamide (**3**), product (**2**) can further reduce to 4-(acetoxyamino)-5-(aziridin-1-yl)-2-nitrobenzamide (**4**) the DNA cross linking species.

Nitroreductases (NTRs) are flavin mononucleotide (FMN) containing enzymes [17,18,19] which have been strongly implicated for a potential role in cancer prodrug therapy due to their ability to reduce organic nitroaromatic compounds such as the prodrug 5-(aziridin-1-yl)-2,4-dinitrobenzamide (CB1954) [20,21,22,23,24]. The CB1954 prodrug undergoes the reduction of one of its nitro groups to hydroxylamine derivatives in the presence of an NTR with NADH or NADPH present as a cofactor [24], the schematic of this reduction is presented in Figure 1. The 4-hydroxylamine derivative from the reduction reacts with intracellular thioesters, such as acetyl CoA, to form a highly cytotoxic product that causes DNA interstrand cross-linking resulting in rapid cell death [8,24]. It is important to note however that the 2-hydroxylamine derivative itself is also highly toxic and exhibits a greater bystander effect than the 4-hydroxylamine but as it does not react to form a DNA cross-linking species it is the less desirable product of the two. Thus, the discovery and development of nitroreductases that preferentially generate the 4-hydroxylamine derivative, rather than the 2-hydroxylamine product, is another area of research that is of growing interest [24,25]. 

The most heavily studied NTR for use in DEPT is NfnB from *Escherichia coli* commonly referred to in the literature as the “bacterial nitroreductase”. The NfnB-CB1954 combination has even progressed to the clinical trial stage using the GDEPT model, with some positive results being seen for both prostate and ovarian cancers [26,27]. Despite some promising results, the fact that the *E. coli* NfnB has an extremely low turnover rate of the prodrug and the highest achievable plasma concentration of CB1954 is less than one eightieth of the lowest reported Km value for *E. coli* NfnB is a major limitation to this research [8,11]. The YfkO nitroreductase from *Bacillus licheniformis* has been shown in the literature to be a promising candidate to move forward within DEPT strategies, due to it having a much higher turnover of CB1954 when compared to NfnB as well as being superior with regard to the efficiency of its reaction with the prodrug [14,28]. 

This aim of this study is to make a direct comparison between the *E. coli* NfnB and the *B. Licheniformis* YfkO in terms of their suitability for use in MNDEPT treatments. We will be directly comparing the kinetic parameters and cell kill potential of the enzymes directly and when immobilized onto AuMNPs in order to assess their suitability for use in MNDEPT [15,29,30].

## 2. Materials and Methods 

All chemicals and reagents were purchased from Sigma Aldrich (Gillingham, UK) unless otherwise stated.

### 2.1. Protein Expression and Purification

A *yfko-cys* or *nfnb-cys* gene (dependent on desired enzyme) that had been previously cloned into the pET28a(+) (Novagen, Merck, UK) expression vector, was transformed into an *E. coli Rosetta* strain B21 DE3 (Novagen, Merck, UK) and the expression and purification of the genetically modified enzymes was performed as previously described [15]. A colony of *E. coli Rosetta* which contained the expression vector with the relevant enzyme gene, was added to a Luria-Bertani (LB) inoculant tube (5 mL) inoculated with kanamycin (50 µg/mL). The inoculant tube was then vortexed overnight at 1500 rpm for 16 h. After this 16 h period the inoculant was added to a flask containing LB expression medium (500 mL) and kanamycin (50 µg/mL). This expression medium was left to grow until it had reached an optical density of 0.6–0.7 measured at 590 nm, after which expression of protein was induced by adding isopropyl-β-d-thio-galactoside (IPTG) (2 mL, 100 mM). The culture was harvested after 4 h of nitroreductase expression by centrifugation (9318 rcf, 10 min, 4 °C). Expressed nitroreductase was released from cells by sonicating the cell pellet in imidazole (10 mm, pH 7.2) for 2 min on ice. The desired enzyme was purified from the cell debris first by centrifugation (44,800 relative centrifugal force (rcf), 1 h) and passing the subsequent supernatant through a metal ion affinity chromatography column using Ni^2+^, with imidazole as the eluent. Eluted protein was incubated on ice for 1 h with flavin mononucleotide to ensure enzyme active site saturation. Impurities were removed from the solution via size exclusion chromatography with a PD10 column using phosphate buffer (50 mM, pH 7.2) as the eluent. A 12% SDS-PAGE was used to assess the purity and weight of the purified proteins, using Coomassie blue as the stain visualizer. The Bradford assay using a BSA standard curve was performed to assess the protein concentration, this was done according to the manufacturer’s instructions.

### 2.2. Enzyme Reactivity with CB1954

The purified enzyme’s reactivity towards the CB1954 prodrug was determined as previously described [15]. Briefly, enzyme (25 mg/mL) was incubated with NADPH (300 μM) and CB1954 (100 μM) in phosphate buffer (PB) (50 mM, pH 7.2) and absorbance spectra (200–800 nm) was measured every 90 s for 15 min on a Jasco V-550, UV-vis spectrophotometer. For active enzyme/CB1954 combinations, product formation was measured at 420 nm [15,31].

### 2.3. CB1954 Kinetics 

CB1954 kinetic experiments were all carried out in a 96-well microtiter plate (Corning, USA) using a Thermoscientific Varioskan 96-well plate microplate reader using the method that we have previously described [32,33,34]. Product formation was measured at 420 nm over time to determine the -Menten kinetic parameters of the CB1954 prodrug with either NfnB-Cys or YfkO-Cys. CB1954 (0.1–10 mM), NADH (400 µM) and PB (50 mM, pH 7.2) were combined and incubated at 37 °C for 3 min before purified NfnB-Cys or YfkO-Cys (10 µg/mL). Hydroxylamine yield per second was determined by calculating the change in absorbance over 20 s and the molar extinction coefficient, which is the same for both products (ε = 1200 M^−1^ cm^−1^ at 420 nm) [12,13,15,25,32]. Data was analysed using SigmaPlot 12 (SPSS, Systat Software Inc., Chicago, USA) where a non-linear regression tool was used to generate a Michaelis-Menten hyperbolic curve and a report containing the important kinetic information of the system under test. Coefficient of Variability (CoV) for the kinetics is 7.06%.

### 2.4. HPLC Analysis

All HPLC experiments were carried out on a UHPLC machine (Dionex Ultimate 3000 HPLC system, ThermoScientific, Waltham, MA, USA) using a C18 column for analysis (Waters Spherisorb^®^ 5 µm ODS2 4.6 mm × 250 mm C18 column, Wilmslow, UK) following the method previously described [31,32]. HPLC runs were performed with the instrument set to the following parameters: 50 µL injection volume, a fixed column oven temperate of 25 °C, a run length of 45 min and the UV wavelength for detection was 420 nm [31,32,34]. Owing to the light sensitive nature of some of the reagents, samples that were designated for HPLC were prepared in 15 mL amber falcon tubes as follows: NADH (120 µL, 10 mM) nitroreductase (116 µg/mL), CB1954 (20 µL, 50 mM) then made to a final volume of 1080 µL using PB (50 mM, pH 7.2). The reaction mixture was incubated at 25 °C for 15 min, and then degassed under nitrogen (g) for 15 min, giving a total reaction time of 30 min. Following the completion of the reaction time, 750 µL of the de-gassed reaction was pipetted into a chromacol select 2 mL vial (2-SVW8-CPK) and placed into the HPLC machine. The solvent ratio used for all HPLC runs was a 10:90 ratio of acetonitrile/water at a ratio, for the first 20 min of the HPLC run the acetonitrile concentration was increased by 1% per minute to a ratio of 30:70 at the 20 min timepoint. Following this 20 min time point the acetonitrile concentration was altered to keep increasing by 40% per minute to reach a concentration of 100% acetonitrile after 22 min. Results were analysed and validated against literature values and standard spectrum scans at 420 nm of CB1954 (20 µL, 50 mM), NADH (120 µL, 10 mM), and nitroreductase (116 ug/mL), spectrum scans can be found within Supplementary Materials. Ratios of the 2′ and 4′-hydroxylamine products were determined at 420 nm, where both products have equal absorbance. CoV for the HPLC is 5.25%.

### 2.5. Cell Viability Assays

Cell viability assays were performed as previously described [32,33,34]. SK-OV-3 (ECACC 91091004) ovarian cancer cells (Sigma–Aldrich, Gillingham, UK) were seeded into a 96-well plate (corning, Corning, NY, USA) at a density of 1 × 10^3^ cells per well, in 100 µL Dulbecco’s Modified Eagles Medium (DMEM) containing 10% Fetal Bovine Serum (FBS), 1% penicillin/streptomycin and 1% l-glutamine, and were left to attach to the plate in a 5% CO_2_ incubator at 37 °C overnight. After 16 h, the medium was carefully aspirated off, and fresh medium containing increasing concentrations of 25 to 200 nM of enzyme or immobilized enzyme (50 µL) was added to the wells along with CB1954 (100 µM). Wells where only enzyme or immobilized enzyme (200 nM), CB1954 (10 µM) or DMEM (100 µL) were added served as controls with the final volume in each wellbeing 100 µL. After 4 h in a CO_2_ incubator at 37 °C, the medium was removed, and cells were replenished with fresh complete DMEM (100 µL). After 48 h, MTT (3-(4,5-dimethylthiazol-2-yl)-2,5-diphenyltetrazolium bromide) (20 µL, 5 mg/mL) was added to each well and incubated at 37 °C for 4 h. Culture medium was carefully aspirated, the purple formazan crystals that formed were then dissolved in Dimethyl sulfoxide (DMSO) (100 µL) and the absorbance was read at 570 nm in a Thermo Scientific Varioskan 96-well plate microplate reader. CoV for the cell culture is 2.76%.

### 2.6. Synthesis and Purification of AuMNPs

Fe_3_O_4_ nanoparticles (220 µL, 3 mg/mL) were added to sodium citrate dihydrate (4.5 mL, 100 mM) in deionized water (145.5 mL) and degassed in argon for 30 min, following degassing the mixture was magnetically stirred for the remainder of the reaction. After the reaction had been stirring for 30 min, Au seeds (1 mL) were then slowly added to the mixture before being left to stir for another hour. HAuCl_4_ (5 mL, 1% by weight) was then added slowly at a rate of 1 mL per minute and left to stir for 5 min, NH_2_OH (1 mL, 200 mM) was then added at 0.5 mL per minute and the reaction was left to stir for an hour at 60 °C. Sodium citrate dihydrate (4.5 mL, 100 mM) was then added and the reaction was stirred for an hour before being removed from the reaction vessel and centrifuged for an hour at 1270 rcf and 20 °C. The supernatant was then extracted from the centrifuge tubes without disturbing the pellet, placed into 8 mL glass vials and magnetically separated using a 50 × 50 × 25 mm 1T Neodymium magnet (First4magnets, Tuxford, UK). The final step of the purification was to centrifuge the AuMNPs at 500 rcf for 2 h to allow full separation of the AuMNPs and any uncoated Fe_3_O_4_ nanoparticles. CoV for the AuMNP synthesis is 26.06%.

### 2.7. Conjugation of Cys-Tagged Enzymes to Au-MNPs

Magnetically purified AuMNPs, suspended in sodium citrate dihydrate (1 mM, pH 7.4), were incubated with a volume of the enzyme at a ratio of 1 AuMNP:270 enzymes to achieve a monolayer coating of the nanoparticles [15]. The volume of enzyme incubated with AuMNPs was determined based upon the concentration calculated using the Bradford assay and the concentration and size of AuMNPs as determined by UV-Vis [15]. Nano-conjugates were left to form at 4 °C for 48 h. A full wavelength UV-Vis scan (450–650 nm) was performed on AuMNPs before and after conjugation, to observe any changes in the gold peak caused by conjugation. A successful conjugation is indicated by a redshift of 3–5 nm of the λ-max of the gold peak [15,35].

### 2.8. Characterisation of AuMNPs and Subsequent Conjugates

Magnetically purified AuMNPs were sent to Cardiff University for TEM analysis to assess particle morphology. Briefly, 5 mL of purified particles, suspended in sodium citrate (1 mM, pH 7.4), were dried down onto formvar/carbon coated 300 mesh grids and examined and imaged in a Philips CM12 TEM (FEI U. K. Ltd., Cambridge, UK) at 80 kV and images captured with a Megaview III camera and AnalySIS software v. 3.1 (Soft Imaging System GmbH, Münster, Germany). Particle size was measured with ImageJ software. Next, AuMNPs and subsequent AuMNP-NTR conjugates were assessed using UV-Vis spectroscopy as per our previous work [15,33]. Briefly, a full spectrum UV-Vis scan was obtained of the synthesised nanoparticles, before and after being conjugated to an enzyme, to observe any changes in the λ-max of the gold peak that would indicate the successful formation of an enzyme monolayer on the particle surface.

Finally, cyclic voltammetry was used to confirm if the binding of the cys-tagged enzymes to a gold surface was occurring via thiol bonds as planned. All measurements were performed using an Autolab PGSTAT 30 computer-controlled electrochemical measurement system (Eco Chemie, Utrecht, Holland). The analysis was carried out with a three-electrode cell; using a saturated calomel electrode (SCE) as the reference electrode and a platinum counter electrode. The working electrode was an enzymatically modified gold coated glass slide. Prior to the formation of the enzyme layer, the gold coated glass slides (Winkler GmbH, Stuttgart, Germany) were flame annealed resulting in a flat gold surface with strong Au (111) characteristics [36]. Two gold slides were then incubated glass back to the glass back in the purified desalted NTR for 48 h at 5 °C. The electrode cell was purged with nitrogen for 20 min prior to running and the cell was placed in the faraday cage and the software was run using the following parameters: scan 0.0 V to −1.2 V at 50 mV/s and scanned twice. The data was then collected and processed using Ecochemie GPES software and presented using Microsoft Excel. 

## 3. Results

### 3.1. Protein Expression and Purification

The recombinant proteins were all purified using metal ion affinity chromatography (IMAC). As observed previously, both cys-tagged enzymes required a higher concentration of imidazole to be eluted than their his-only counterparts [15]. The purity and molecular weight of each protein was determined before use in further experiments. Molecular weights of the proteins were established using SDS-PAGE and protein concentrations were determined using the Bradford method and expressions generally yielded 2–5 mg/mL pure protein.

### 3.2. Characterization of AuMNPs and Subsequent Conjugates

**Figure 2:**
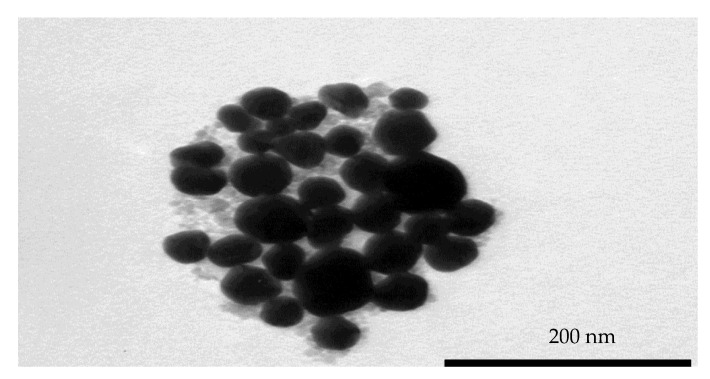
A TEM image of synthesized, magnetically purified, uncoated AuMNPs.

A TEM image of the synthesis and purified AuMNPs was obtained, and it was established that the sample contained predominately AuMNPs with a small amount of uncoated Fe_3_O_4_ the sample (Figure 2).

The figure shows that the particles are spherical in shape and have an average size of approximately 50 nm, with a standard deviation of ±11 nm from a sample size of 30 nanoparticles. As can be seen in the figure, there is a very low count of uncoated Fe_3_O_4_ nanoparticles from the sample that had yet to undergo final centrifugation purification.

**Figure 3:**
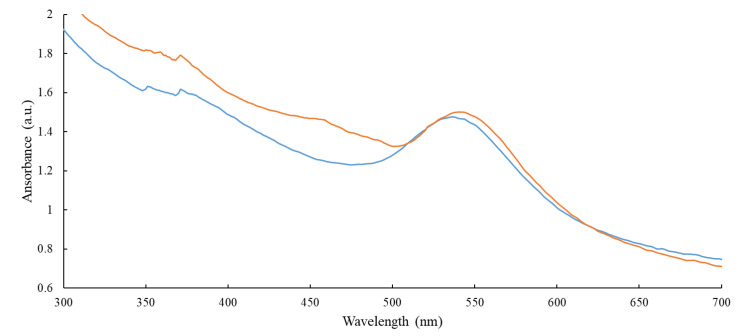
An overlay of the full spectrum UV-Vis scans obtained for AuMNPs, before and after conjugation to an NTR. The blue line represents the AuMNPs prior to conjugation (λ-max = 536 nm) and the orange line represents the AuMNP-NTR conjugate (λ-max = 540 nm).

Next, AuMNPs were conjugated with either NfnB-cys or YfkO-cys at a ratio of 1:270, as per our previous published work [15]. A full spectrum UV-Vis scan was performed before and after conjugation to look for any changes in the λ-max of the gold peak (Figure 3). 

Figure 3 is an overlay of the scans obtained before and after conjugation, where the blue line represents AuMNP and the orange line represents AuMNP immobilised YfkO-cys. The λ-max of the gold peak prior to conjugation is 536 nm. Then, once enzymes are conjugated onto the surface of the nanoparticles, there is a redshift in the λ-max to give a new value of 540 nm. This indicates that the enzymes have successfully immobilised onto the particles, as the λ-max of gold nanoparticles is directly correlated to the overall size of the nanoparticle, therefore by increasing the size by conjugating enzymes onto the surface of the nanoparticle the overall λ-max of the UV-vis spectra will increase [15]. YfkO-cys is presented here as an example, but the same results were observed when using NfnB-cys.

**Figure 4:**
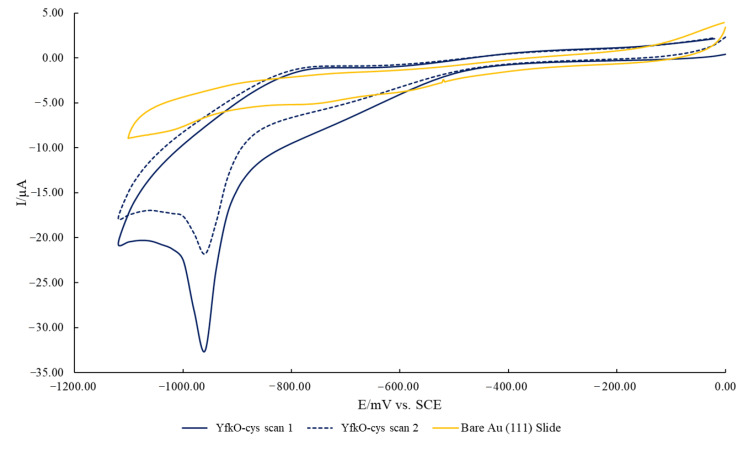
Cyclic voltammogram showing a reduction cycles of a 70 bare Au(111) coated glass slide and two consecutive reduction cycles of an Au(111) coated glass slide after 48 h incubation with YfkO-cys: scan 1 (solid line) = −959.47 mV, −32.60 µA and scan 2 (dashed line) = −959.47 mV, −21.82 µA. The potential is vs. SCE in an electrolyte of 0.1 M NaOH.

Next, the nature of the binding of the cys-tagged enzymes to a gold surface was evaluated using cyclic voltammetry, based on the change in the gold reduction peak after immobilisation (Figure 4).

The voltammogram exhibits the characteristic cathodic peak at roughly 980 mV that is associated with thiol desorption [37]; thereby confirming that the cys-tagged enzymes were successfully bound to the surface of the gold surface via thiol bonds. This allows us to conclude that the enzymes were also immobilised onto the surface of the gold-coated iron nanoparticles via thiol bonds. Again, while YfkO-cys has been shown as the example here, the same results were also seen for NfnB-cys. 

### 3.3. Enzyme Reactivity with CB1954

**Figure 5:**
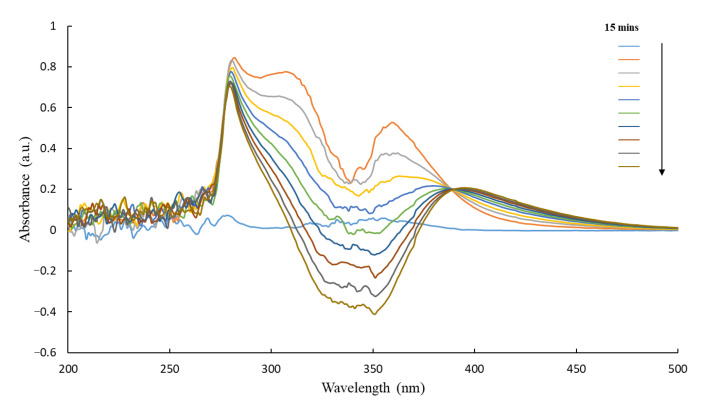
UV-Vis spectra of YfkO-cys showing the enzymatic activity assay with increasing time. (1) NTR absorbance ~290 nm. (2) Reduction of CB1954 λ-max = 327 nm. (3) Oxidation of NADH λ-max = 340 nm. (4) Production of CB1954 hydroxylamine derivatives λ-max = 420 nm.

Once purified, the proteins were tested for their reactivity towards CB1954 and ability to reduce its nitro groups. Active proteins would be able to reduce CB1954 to either the 2- or 4-hydroxylamine derivatives in the presence of a cofactor, which in this case was NADH. This could be monitored using UV-Visible spectroscopy as with each scan for an active protein the peak corresponding to NADH would decrease (λ-max = 340 nm) and the peak corresponding to the CB1954 hydroxylamine derivatives would increase (λ-max = 420 nm) (Figure 5).

**Figure 6:**
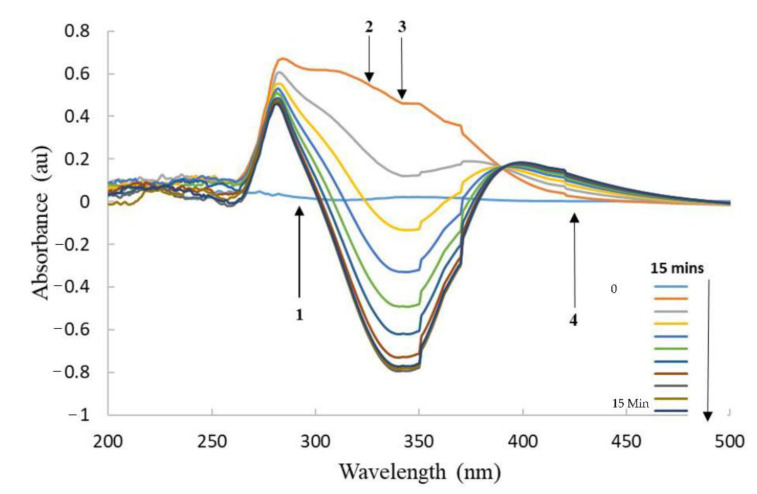
UV-Vis spectra of AuMNP immobilised YfkO-cys showing the enzymatic activity assay with increasing time. (1) NTR absorbance ~290 nm. (2) Reduction of CB1954 λ-max = 327 nm. (3) Oxidation of NADH λ-max = 340 nm. (4) Production of CB1954 hydroxylamine derivatives λ-max = 420 nm.

All enzymes were shown to be active with the CB1954 prodrug when tested in this way. Furthermore, all enzymes retained their activity when immobilised to the Au-MNPs (Figure 6).

The UV-Vis activity scans for YfkO-cys have been presented as an example here, but the scans obtained for NfnB-cys, free and immobilised, were almost identical to these.

### 3.4. Michaelis-Menten Kinetics

Using the hyperbola regression analysis tool in SigmaPlot, the Michaelis-Menten kinetic parameters with respect to varying concentrations of CB1954 at 37 °C were established using the absorbance of the CB1954 hydroxylamine derivatives at 420 nm (Table 1). This was done for both free and immobilised YfkO-cys and compared to our previously published data for free and immobilised NfnB-cys [32].

When comparing the Michaelis-Menten kinetic data obtained for free and immobilised YfkO-cys with the data obtained previously, there are several interesting observations. Firstly, when comparing the two enzymes free in solution, it is evident that YfkO-cys is much more efficient in its reaction with the CB1954 (Kcat/Km = 0.0471 µM^−1^ s^−1^ for YfkO-cys compared to 0.0109 µM^−1^ s^−1^ for NfnB-cys) which is four times greater in efficiency where Kcat is the rate of catalytic conversion and Km is ½ of Vmax which is, itself the maximum rate of the system. This was predominately caused by the fact that YfkO-cys has a much higher affinity for the CB1954 prodrug as evidenced by the lower Km (Km = 834.33 µM for YfkO-cys compared to 5078.37 µM for NfnB-cys). The next notable finding is that, unlike previously for NfnB-cys [32], when YfkO-cys was immobilised onto the nanoparticles it became less efficient in its reaction with the CB1954 (Kcat/Km = 0.0366 µM^−1^ s^−1^ for free YfkO-cys compared to 0.0471 µM^−1^ s^−1^ for immobilised YfkO-cys). This is because, despite the fact that immobilised YfkO-cys demonstrated a higher turnover of the CB1954 (Kcat = 39.34 s^−1^ for free YfkO-cys compared to 50.30 s^−1^ for immobilised YfkO-cys), the Km increased by more than half once it was immobilised onto the nanoparticles (Km = 834.33 µM for free YfkO-cys compared to 1374.59 µM for immobilised YfkO-cys).

### 3.5. HPLC of CB1954 Products

Following the method we have previously described [32] the CB1954 hydroxylamine product ratio was assessed for free and immobilised YfkO-cys and compared to the previously published results for free and immobilised NfnB-cys, these values can be seen below in Table 2 [32].

The hydroxylamine product ratios produced by free (4:96) and immobilised (1:99) YfkO-cys were both extremely similar to one another, and in both cases, the enzyme demonstrated a preference for reducing CB1954 at the 4-nitro position. This result is of clinical significance because the 4-hydroxylamine product has been shown in the literature to be further reduced by intracellular thioesters, like Acetyl Coenzyme A, to form a compound able to cross-link DNA [8,12,24].

### 3.6. Cell Viability Assays

**Figure 7:**
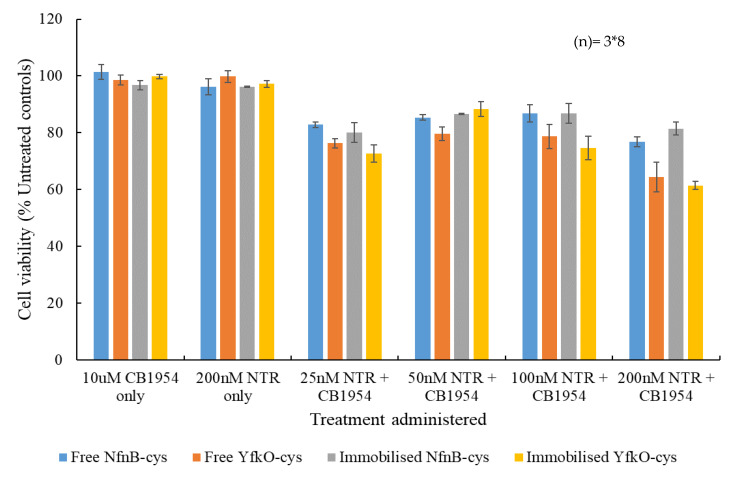
Percentage cell survival relative to untreated control cells of SK-OV-3 cells after a 4 h incubation with prodrug only, enzyme only and increasing concentrations of either free or immobilised NfnB-cys or YfkO-cys (25–200 nM) in presence of CB1954 (10 µM). All data points are taken from the averages of at least three repeats and the error bars represent the standard deviation. Data points were analysed for their statistical significance using F-test with a p value of (>0.005), error bars are ±1 standard deviation, and (*n*) is the number of repeats each data point had.

Percentage cell viability assays were carried out using the ovarian cancer cell line, SK-OV-3, relative to untreated controls, in the presence of increasing concentrations of free or immobilised NfnB-cys or YfkO-cys, with a constant concentration of CB1954 added (Figure 7). The controls used were cell culture medium only, the enzyme only and prodrug only, and data points were plotted based on the averages of at least three repeats. The CB1954 concentration was fixed at 10 µM so that the results of the cell viability assays could be related to clinical results. The maximum concentration of CB1954 achievable in vivo cannot exceed 10 µM, due to the maximum tolerated dose established during clinical trials [21,38,39].

Promisingly, no significant cell killing was observed when the cells were treated with either the prodrug alone or the enzyme alone. Upon being treated with the lowest concentration of enzyme (25 nM) plus prodrug, there was an immediate response for all combinations—with cell viabilities between 70% and 80%. The data was analysed for statistical significance by F-test with all data sets demonstrating levels of statistical significance (*p* < 0.005). Immobilised YfkO-cys produced the most potent response at this concentration, whereas free NfnB-cys produced the weakest response. For all combinations, there was an initial decrease in the cell killing potency when the enzyme concentration was increased from 25 to 50 nM, before the cell killing potency increased again as the enzyme concentration approached 200 nM. Whilst this appears to be contrary to what might be expected, it could be attributed to a well-documented effect, known as the Hormetic effect [40]. The Hormetic effect is evident from the characteristic inverted- U-shaped response on the graph [40], and it is a phenomenon that we have come to expect when testing CB1954’s cell killing potency in SK-OV-3 cells [32,33,34].

## 4. Discussion

The major aim of this study was to assess the YfkO nitroreductase as an alternative to the heavily investigated NfnB nitroreductase for use in MNDEPT treatments in terms of their ability to reduce the CB1954 prodrug and cause cell death in cancerous cell lines when immobilised on to the Au-MNPs. Furthermore, it was also hoped that the genetically modified YfkO-cys NTR would produce a more favourable ratio of the CB1954 hydroxylamine derivatives and be superior in terms of its kinetic activity with CB1954 when compared to the corresponding NfnB. 

Gold-coated Iron Nanoparticles (AuMNPs) were successfully synthesized and purified, and their size was confirmed to be roughly 50 nm in size using TEM. We have shown in previous work that AuMNPs of this size can be directly uptaken into cancerous cells via endocytosis [33], so this is significant because it means that, if we could form active AuMNP-NTR conjugates, we would have a prodrug-activating system capable of being uptaken into cancerous cells where it could access intracellular cofactor and facilitate the production of the toxic CB1954 products.

The genetically modified cys-tagged enzymes were designed previously [15] with intention of immobilizing them directly onto the surface of the AuMNPs for use in MNDEPT strategies, so we then needed to test if we were able to successfully form AuMNP-NTR conjugates.

It is well established from previous work [15] that when you immobilize enzymes onto the surface of nanoparticles, increasing the overall size, there is a red-shift in the λ-max of the gold peak. In this work, when immobilizing YfkO-cys onto the nanoparticles, we observed a red-shift of 4 nm, indicating successful conjugation.

Next, we had to establish if the conjugation was via thiol-bonds as intended. Cyclic voltammetry was used to analyse the cys-tagged enzymes that had been immobilised onto gold slides to see if they formed thiol bonds, based on the change in the gold reduction peak after immobilisation. The voltammogram exhibited the characteristic cathodic peak at roughly 980 mV that is associated with thiol desorption onto a gold surface [37]. Based on this, we concluded that enzymes had been immobilised to the AuMNPs via thiol bonds. This is an important result because the cys-tags have been placed on the N-terminus of the enzyme deliberately so that the active site of the enzyme remains accessible by substrates once immobilised [15].

After analysing all the enzymatic activity scans, it was clear that YfkO-cys was able to readily reduce CB1954 to its hydroxylamine derivatives in the presence of NADH as a cofactor, whether the enzyme was free in solution or immobilised onto the nanoparticles. Again, the fact that the enzyme remains active when immobilised, is further evidence of the formation of thiol bonds onto the surface of the AuMNPs because it shows immobilization took place away from the active site, as intended. 

The kinetic data obtained for YfkO is not in agreement with the data reported in the literature due to the fact that this data set was generated using NADH as the cofactor whereas Prosser et al. [14] reported Kcat/Km = 0.025 μM^−1^·s^−1^ for this enzyme using NADPH as the cofactor. NADH was chosen as the cofactor for this data set because it is much more abundant intracellularly than NADPH [41] and it allowed a more direct comparison to the data previously generated for the corresponding NfnB [32]. Despite the differences in values for Kcat/Km, the data reported in this study does agree with the literature in the sense that the reported Kcat/Km for YfkO is greater than that of NfnB free in solution, indicating that the reaction with CB1954 is more efficient when using this enzyme. Unfortunately, the YfkO enzyme did not remain more efficient than NfnB when immobilised onto the AuMNPs. However, the YfkO/CB1954 combination is still likely to perform better in a clinical setting than the NfnB/CB1954 combination because the YfkO enzyme reduces CB1954 almost exclusively at the 4-nitro position, meaning that it will produce much more of the DNA-reactive species that have been identified in previous literature [8,12,24]. Alternatively, some literature has suggested that there are benefits to using enzymes which favour the reduction of CB1954 at the 2-hydroxylamine position because the products formed from that reaction have a higher bystander effect than those formed after reaction at the 4-hydroxylamine position [21,42,43].

Cell viability assays carried out by Vass et al. on the SK-OV-3 cell line appeared to show superior results in terms of the cell viability after treatment compared to the data reported in this study [44]. An explanation for this difference in reported cell viability is that, in their work, they incubated their cells with the additional cofactor (NAD(P)H) whereas in this dataset only the intracellular cofactor is used, with no additional cofactor being added as this gives a better indication of how the treatment will perform in vivo [32,33,34]. The fact that a killing of the cells was observed indicates that the treatment was successfully up taken into the SK-OV-3 cells, where it could access the intracellular cofactor to produce toxic products and induce cell death. It is promising that, of all combinations tested, immobilised YfkO-cys performed the best at the lowest concentration of enzyme tested and this is likely because it almost exclusively reduced CB1954 at the 4-hydroxylamine position, leading to the production of the highly potent DNA cross-linking species. The Anova test was used to asses if each overall set of cell viability contained significant data using a significance level (*p*-value) of 0.005 (99.5% confidence level), the F-value for each set of cell culture data as larger than the f_critical_-value produced by the Anova test, indicating statistically significant results.

## 5. Conclusions

In conclusion, YfkO-cys and NfnB-cys have both been shown to be active with the CB1954 prodrug. Furthermore, both enzymes were successfully immobilised onto the novel AuMNP prodrug-activating enzyme delivery system that we have developed in-house and retained their activity with the prodrug once immobilised. YfkO-cys has been identified as a promising candidate for use in DEPT treatments using CB1954 as it has been shown to reduce CB1954 predominately at the more desirable 4-hydroxylamine position, which leads to the formation of DNA-reactive species when reacted with intracellular thioesters. The next stage of this research will be to use immobilised YfkO-cys in combination with cell-penetrating peptides in 3D cell culture models, as we have shown previously that these can increase the cellular uptake of nanoparticle based treatments [33]. We expect that this will see the cell killing potency of the YfkO-cys/CB1954 increase drastically as more of the enzyme will have access to the intracellular cofactor present within the cells, leading to the formation of more of the toxic CB1954 products.

## Figures and Tables

**Table 1:** Michaelis-Menten data obtained for free and immobilised YfkO-cys with varying concentrations of CB1954, compared to the data we have published previously for free and immobilised NfnB-cys [32].

Enzyme	Vmax (µM s^−1^)	Km (µM)	Kcat (s^−1^)	Kcat/Km (µM^−1^ s^−1^)
NfnB-cys	19.4 ± 1.3	5000 ± 700	55.34 ± 0.57	0.0109 ± 3.8 × 10^−3^
Immobilised NfnB-cys	10.89 ± 1.2	1108 ± 300	61.85 ± 0.64	0.0558 ± 3.5 × 10^−3^
YfkO-cys	6.5 ± 1.2	830 ± 250	39.34 ± 0.89	0.0471 ± 3.6 × 10^−3^
Immobilised YfkO-cys	8.35 ± 1.4	1374 ± 400	50.30 ± 0.78	0.0366 ± 3.7 × 10^−3^

**Table 2:** The ratio of hydroxylamine derivatives formed upon reacting free and immobilised YfkO-cys, compared to those we have previously published for NfnB-cys [32].

Enzyme	Hydroxylamine Product Ratio (2-NHOH:4-NHOH)
NfnB-cys	32:68
Immobilised NfnB-cys	13:87
YfkO-cys	4:96
Immobilised YfkO-cys	1:99

## Data Availability

Data is contained within the article or supplementary material. The data presented in this study are available in article or Supplementary Material.

## Supplementary Materials

The following are available online at https://www.mdpi.com/article/10.3390/pharmaceutics13040517/s1, Figure S1: HPLC chromatogram for YfkO-Cys, Figure S2: HPLC chromatogram for NfnB-Cys, Figure S3: HPLC chromatogram for Immobilized YfkO-Cys, Figure S4: HPLC chromatogram for Immobilized NfnB-Cys, Figure S5: HPLC of a CB1954 standard, Figure S6: HPLC of a nitroreductase standard, Figure S7: HPLC of a NADH standard, Figure S8: HPLC of a NAD+ standard.
