# Supplementary material for: The YfkO Nitroreductase from Bacillus Licheniformis on Gold-Coated Superparamagnetic Nanoparticles: Towards a Novel Directed Enzyme Prodrug Therapy Approach
Source: Pharmaceutics. 2021 Apr 9;13(4):517. doi: 10.3390/pharmaceutics13040517 (PMC8070144; doi:10.3390/pharmaceutics13040517)
Supplement: Supplementary file 1 [file pharmaceutics-13-00517-s001.pdf]

pharmaceutics

MDPI

Supplementary Materials: The YfkO Nitroreductase from Bacillus Licheniformis on Gold-Coated Superparamagnetic Nanoparticles: Towards a Novel Directed Enzyme Prodrug Therapy Approach

Patrick Ball [1], Robert Hobbs [1], Simon Anderson [1], Emma Thompson [1], Vanessa Gwenin [1], Christopher Von Ruhland [2] and Christopher Gwenin [1,3,*]

Publisher's Note: MDPI stays neutral with regard to jurisdictional claims in published maps and institutional affiliations.

Copyright: © 2020 by the authors. Submitted for possible open access publication under the terms and conditions of the Creative Commons Attribution (CC BY) license (http://creativecommons.org/licenses/by/4.0/).

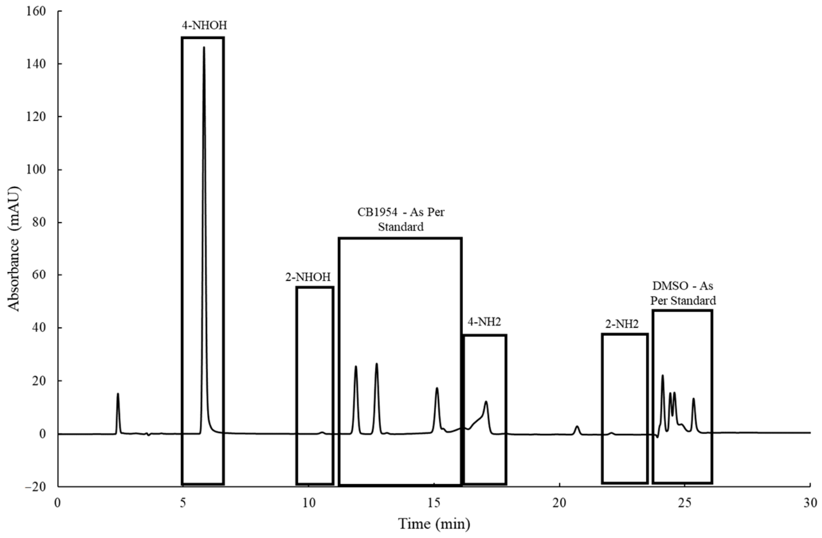

Figure S1. HPLC chromatogram for YfkO-Cys.

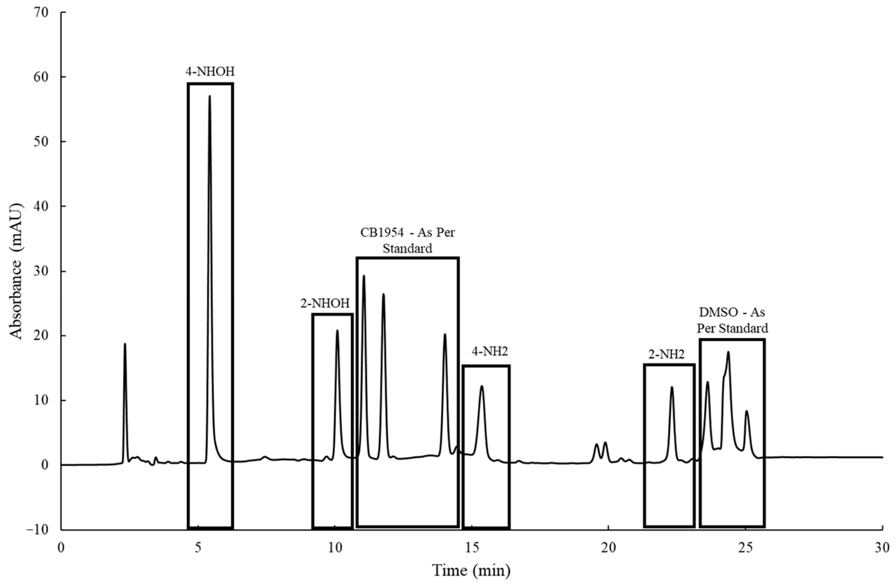

Figure S2. HPLC chromatogram for NfnB-Cys.

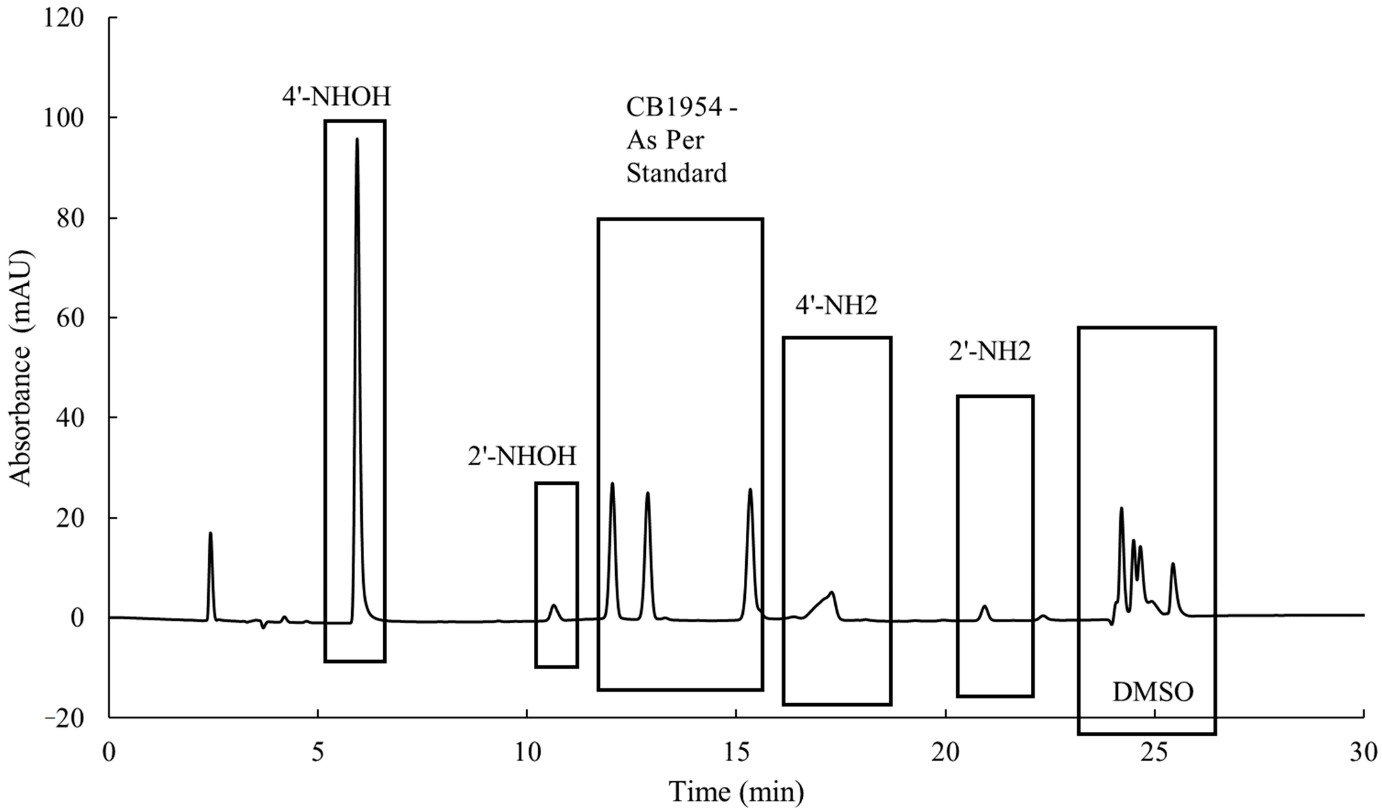

Figure S3. HPLC chromatogram for Immobilized YfkO-Cys.

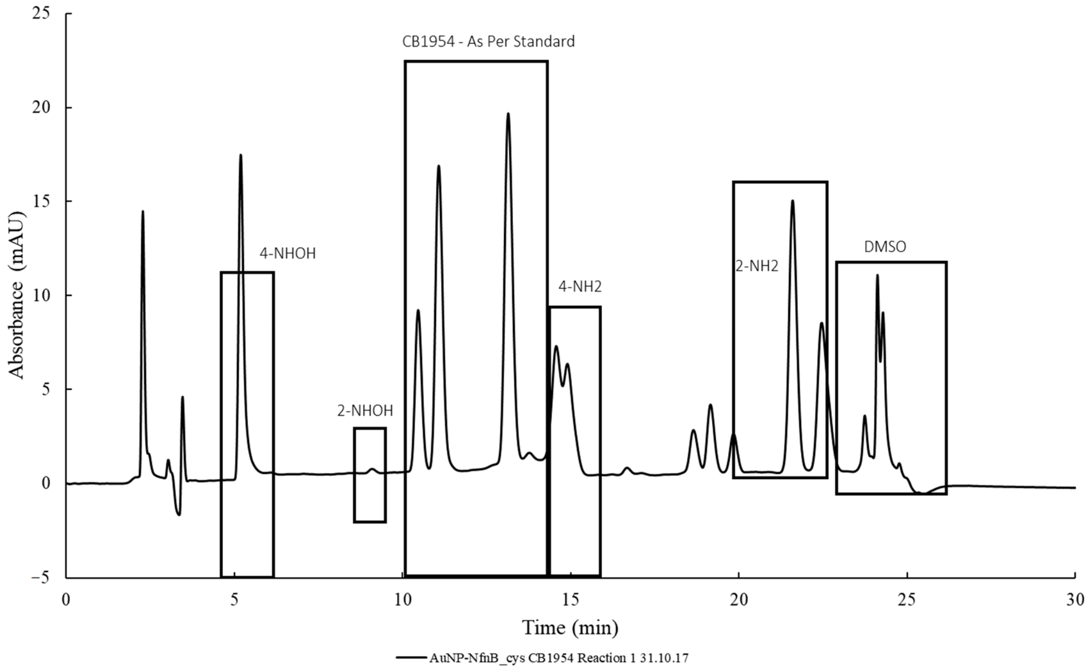

Figure S4. HPLC chromatogram for Immobilized NfnB-Cys.

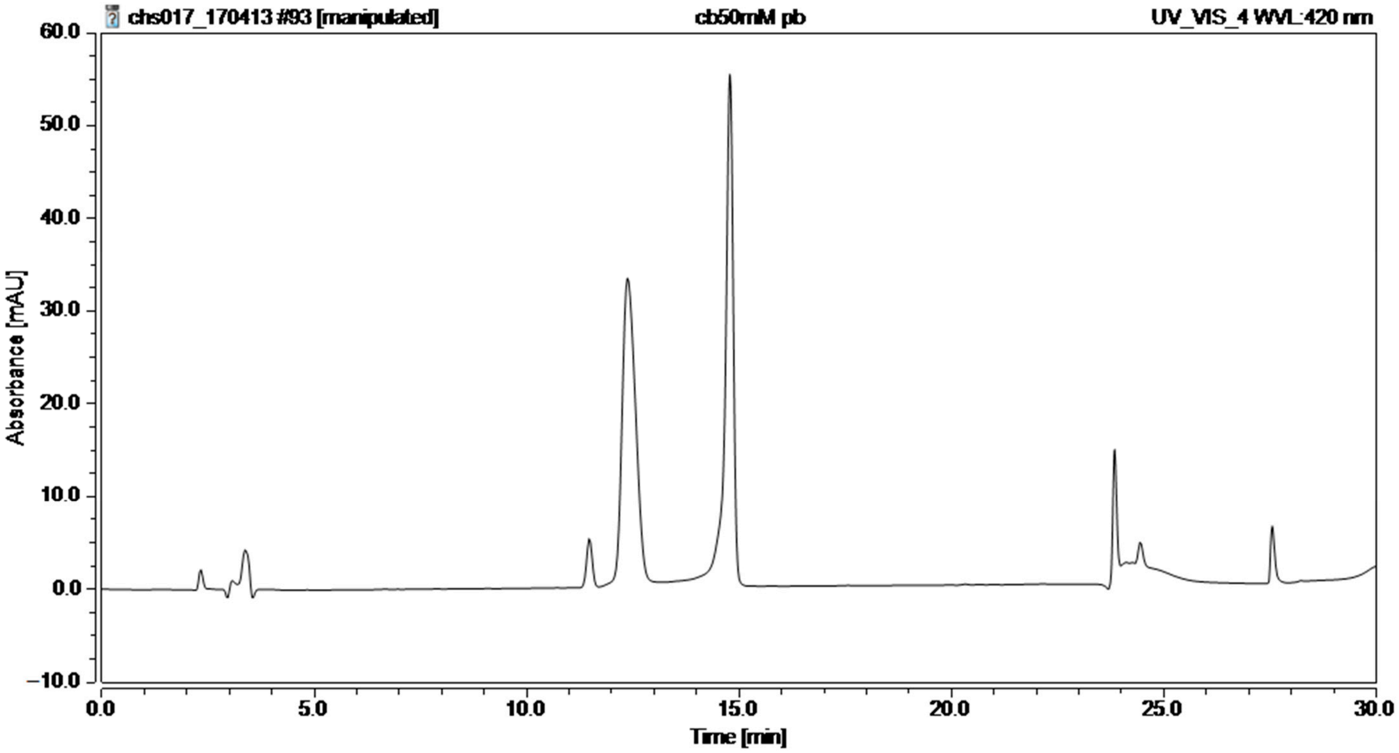

Figure S5. HPLC of a CB1954 standard.

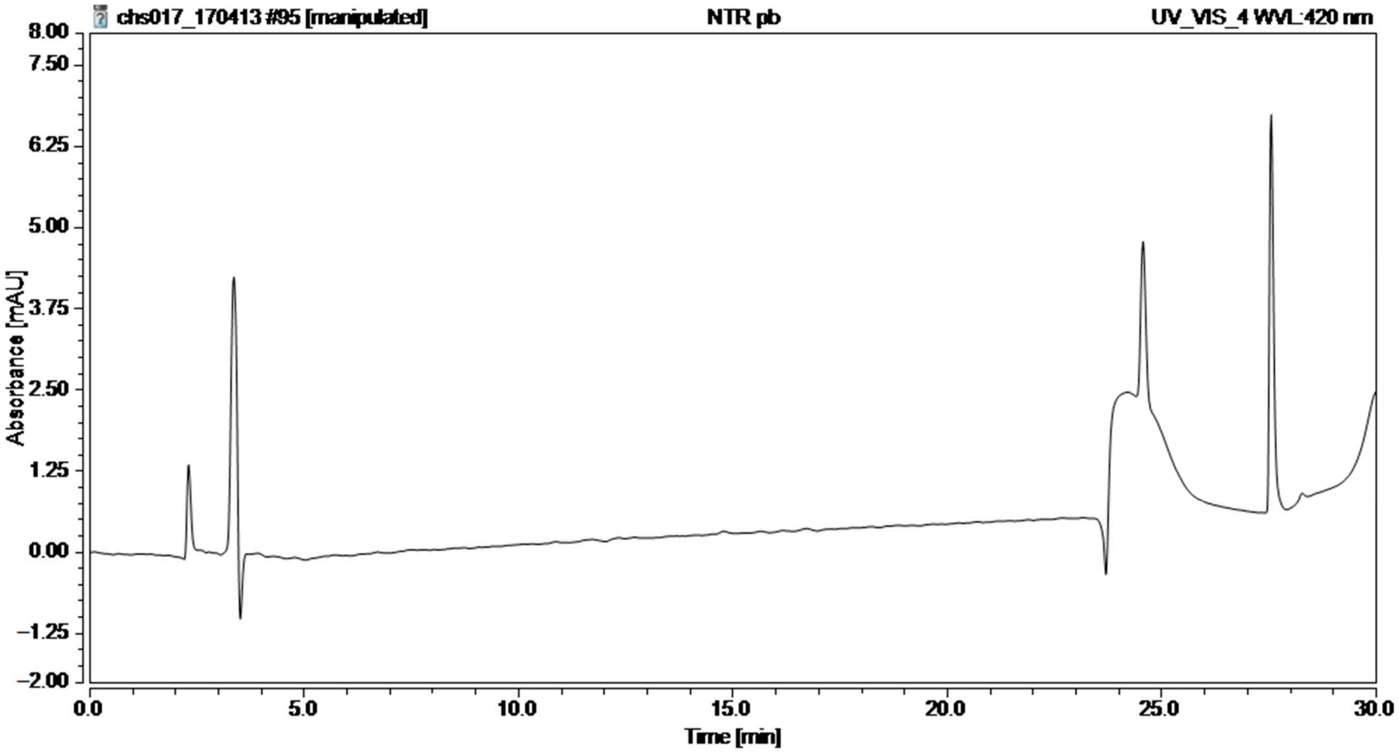

Figure S6. HPLC of a nitroreductase standard.

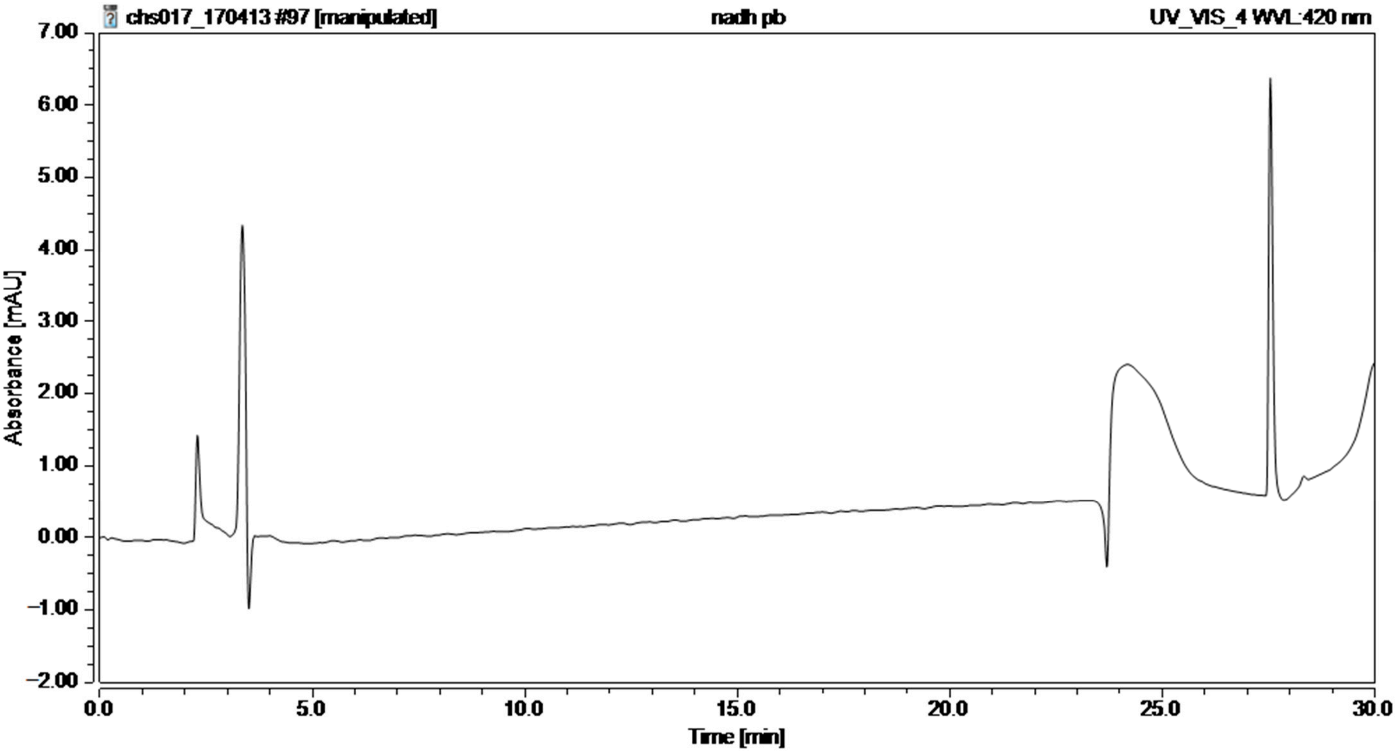

Figure S7. HPLC of a NADH standard.

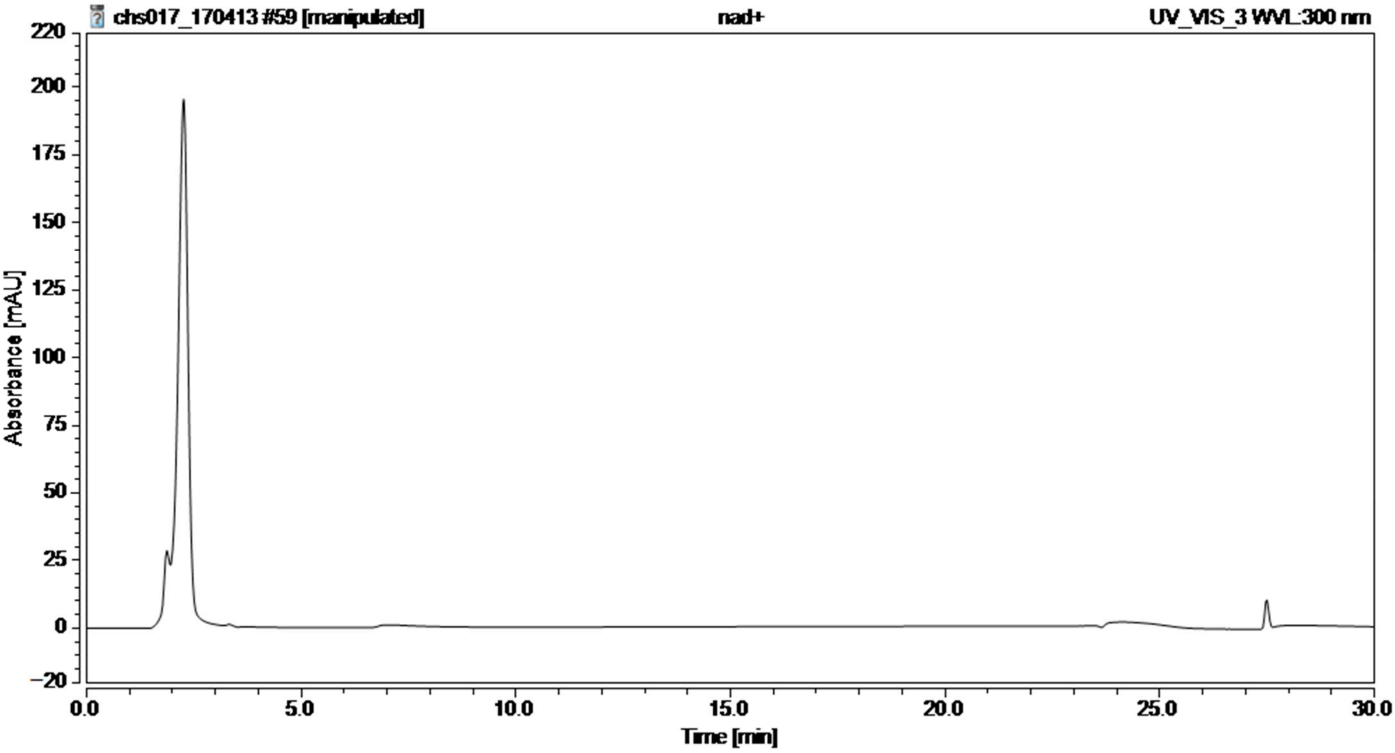

Figure S8. HPLC of a NAD+ standard.
